# Managing Emergencies in Rural North Queensland: The Feasibility of Teletraining

**DOI:** 10.1155/2018/8421346

**Published:** 2018-04-23

**Authors:** Tarsh Pandit, Robin A. Ray, Sabe Sabesan

**Affiliations:** ^1^College of Medicine and Dentistry, James Cook University, Townsville, QLD, Australia; ^2^Tropical Centre for Telehealth Practice and Research, Townsville Hospital and Health Services, Townsville, QLD, Australia

## Abstract

**Introduction:**

Historically, the use of videoconference technologies in emergency medicine training has been limited. Whilst there are anecdotal reports of the use of teletraining for emergency medicine by rural doctors in Australia, minimal evidence exists in the literature. This paper aimed to explore the use of teletraining in the context of managing emergency presentations in rural hospitals.

**Methods:**

Using a qualitative approach, a mixture of junior and senior doctors were invited to participate in semistructured interviews. Data were transcribed and analysed line by line. Applying the grounded theory principles of open and axial coding, themes and subthemes were generated.

**Results:**

A total of 20 interviews were conducted with rural doctors, rural doctors who are medical educators, and emergency medicine specialists. Two major themes—(1) teletraining as education and (2) personal considerations—and ten subthemes were evident from the data. Most participants had some previous experience with teletraining. Access to peer teaching over videoconference was requested by rural generalist registrars. There was a preference for interactive training sessions, over didactic lectures with little mention of technical barriers to engagement. The ability of teletraining to reduce professional isolation was a major benefit for doctors practicing in remote locations.

**Discussion:**

For these rural doctors, teletraining is a feasible method of education delivery. Wider application of teletraining such as its use in peer teaching needs to be explored. The benefits of teletraining suggest that teletraining models need to be core business for health services and training providers, including specialist colleges.

## 1. Introduction

Addressing the rural workforce shortages in Australia has been a challenge for the Australian Government for over 20 years [1]. Issues such as lack of professional contact with colleagues, specialist backup in emergencies, and remoteness from families have been well described in the literature as reasons for doctors leaving rural practice [2]. Further training has also been linked with promoting retention of rural doctors [3]. Given the vast distances in Australia, it is not feasible for doctors to regularly travel to larger centres for upskilling. Whilst some rural sites have sufficient medical staff to conduct regular on-site teaching, this is not feasible for smaller hospitals.

The use of videoconference technologies for medical education (teletraining) has been described in the literature from as early as 1996 [4]. It has successfully been used for paediatric cardiology and surgical education including practical training in laparoscopic procedures [5–7]. In oncology, telesupervision and teleeducation for remote administration of chemotherapy have been successfully implemented in rural Australia [8]. However the application for videoconferencing for emergency medicine (EM) education has been limited [9]. Encouragingly a recent Canadian study demonstrated that videoconference technologies can be successfully used to teach the internationally recognized Advanced Trauma Life Support course [10].

A state-wide roll-out of telehealth services by Queensland's Department of Health has resulted in rural doctors having access to high quality videoconferencing technology [11]. Both web based applications and traditional high-end videoconferencing capabilities have been installed in many rural hospitals as part of this state-wide agenda. Anecdotal evidence suggests that videoconferencing is regularly used for EM training in North Queensland, such as the Rural Grand Round and lectures delivered by the Emergency Medicine Education and Training (EMET) team. However, the literature is limited in many aspects of teletraining including description of training models and needs of the rural doctors.

This study aimed to evaluate the training needs of rural doctors and explore the use of teletraining in the context of managing emergency presentations. For the purposes of the paper, only the findings relevant to the use of teletraining have been reported.

## 2. Methods

### 2.1. Recruitment

Using purposive and theoretical sampling, doctors with range of practice years were invited by telephone, email, and professional encounters to participate in the study.

### 2.2. Participants

The inclusion criteria were doctors working in North Queensland with (a) current/recent work in a rural area that involved managing emergencies, (b) involvement with rural medical education, or (c) Fellowship of the Australasian College of Emergency Medicine and working in a tertiary referral centre. Rurality was defined as working in a Modified Monash (MM) category greater than 3. The seven Modified Monash Model categories are a revised version of the previous Australian Standard Geographic Classification-Remoteness Areas (ASGC-RA) which add an extra dimension of population size: (1) metropolitan; (2) regional > 50,000; (3) large rural 15,000–50,000; (4) medium rural 5000–15,000; (5) small rural < 5000; (6) remote; (7) very remote [12, 13].

Roughly equal numbers of junior and senior doctors were recruited. Senior doctors were defined as those who had obtained full fellowship of their respective college, and junior doctors were defined as any doctor yet to meet this criterion. (Fellowship of the Australasian College of Emergency Medicine requires a minimum of 5 years of postgraduate training and satisfactory progression through required assessments. Fellowship of the Australian College of Rural and Remote Medicine or Royal Australian College of General Practitioners requires a minimum of 4 years of postgraduate training and satisfactory progression through required assessments.)

### 2.3. Interview Guide

Adopting a descriptive qualitative approach, an interview guide based on current literature was developed by the research team and revised after discussion with three rural doctors and the approving Human Research Ethics Committee (HREC) [14]. The guide was piloted and refined for data collection. Semistructured interviews covering topics such as practice demographics, experience with video-conferenced based technologies, positive and negative aspects of teletraining, and feasibility of teletraining were conducted by the first author.

### 2.4. Data Analysis

Data analysis occurred concurrently with data collection. Interviews were audiorecorded, transcribed, and analysed line by line in NVivo data management software version 11©. To improve analytical rigour a selected number of interviews were descriptively coded independently by two investigators. The codes were compared to ensure consistency and any disagreements between coding were resolved by consensus. As new themes arose from the analysis, the interview guide was amended enabling a process of participant validation to further enhance the validity of the data. Applying the grounded theory principles of open and axial coding, themes and subthemes were generated. Data saturation was achieved when no new themes emerged in the last four interviews. Relationships between themes were explored using mind-mapping. Demographic information such as position, qualification, and MM category was incorporated into the analysis. Ethical approval to conduct the study was granted by Queensland Health and James Cook University.

## 3. Results

A total of 20 interviews averaging 28 minutes were conducted with doctors from various rural hospitals in North Queensland. Junior doctors were two rural relievers, two GP registrars working as primary house officers, one non-hospital based GP registrar, and six provisional fellows. Senior doctors were seven rural generalists and two emergency medicine specialists (see Table 1 for participant characteristics).

Ten interviews were conducted in person, nine were conducted via telephone and one was conducted over videoconference. During one interview the participant withdrew midinterview due to a clinical emergency. Attempts were made to reschedule, but this was not feasible due to the participant's work schedule.

*Two Major Themes*. Teletraining as education and personal considerations alongside ten subthemes arose from the data.

### 3.1. Teletraining as Education

#### 3.1.1. Previous Experiences with Teletraining

Most participants reported previous experience with teletraining. These experiences included tutorials provided by GP training organisations, lectures by EMET, and training on nonemergency topics such as sexual and mental health in primary care. Two participants mentioned undertaking further academic studies that used videoconference for lectures.

All participants exposed to teletraining talked about its use in positive terms. In particular the Rural Grand Round, a video-conferenced presentation between multiple rural sites, was rated highly by a number of participants. The ability for further education with specialists of the region was also beneficial to rural doctors (see Table 2).

However, some participants described negative experiences in relation to relevance of presentations to current working conditions, sessions lacking interaction, or presentation skills of the presenter. Interestingly, those who described negative experiences continued to recognize it as an acceptable mode of education delivery.

#### 3.1.2. Face-to-Face versus Videoconference Learning

Overall participants perceived teletraining to be a good adjunct to traditional forms of teaching for staff of all levels. The ability to have regular, ongoing education without significant travel was a key factor for doctors, particularly in high MM categories (6 and 7). Participants with significant previous experience with teletraining viewed it as a regular part of continuing education. Whilst some participants mentioned that it was not as good as face-to-face training, they agreed that it was preferable to no training at all.

#### 3.1.3. Peer Teaching

Peer teaching using videoconference was supported by a number of provisional fellows and rural generalists. It was suggested that this teaching should be an interactive, case-based small group discussion facilitated by a senior doctor. The ability to share different approaches relevant to rural settings, formalising conversations that already occur informally, and having training targeted to the participant's level were cited as positive reasons to provide peer teaching.

#### 3.1.4. Teaching Skills Using Videoconference

Concerns about teaching skills over videoconference ranged from technological challenges such as insufficient camera angles to correct technique and the notion that teletraining should only be used for static forms of learning and that certain skills could only be learnt by feeling the anatomy.

However, of the 20 doctors, only one participant reported learning skills in a setting similar to videoconferencing where a colonoscopy was broadcast on a screen to viewers outside the procedure room. The participant suggested that similar demonstrations may be useful.

In terms of learning new skills, participants varied in their opinions about which skills could be successfully taught over videoconference. For example, some doctors stated advanced airways skills could not be taught over videoconference, whilst others suggested it would be achievable if combined with simulation training. There was further disagreement between some junior and senior doctors about the addition of videoconferencing to simulations. The senior doctors argued that technologically intensive simulations detracted from the learning experience; therefore local staff should be trained to run the simulations. However, the suggested benefit of the addition of videoconference to simulations was organisation by an experienced external source, thus making training less likely to be missed.

For brushing up on previous learnt skills, teletraining was not seen to be useful, due to the availability of online resources such as YouTube™ or Life in the Fast lane©. The ability to use resources anytime, when required, was preferable to real-time videoconference education.

#### 3.1.5. Didactic versus Interactive Sessions

Having an interactive component, particularly the ability to have a back-forth discussion is an important aspect of learning via teletraining. Whilst some participants agreed that didactic sessions could be useful in certain scenarios such as major updates, this information could also be provided through online sources. The overwhelming preference was for interactive sessions with increased engagement in discussion sessions.

### 3.2. Personal Considerations

#### 3.2.1. Technological Issues

Interestingly many participants reported minimal issues with Internet connections. Of the doctors who mentioned issues with Internet connections, most described it as a potential challenge rather than a continual problem or only in relation to home Internet. However one doctor did report a number of experiences of technological issues that detracted from the learning experience (see Table 3).

#### 3.2.2. Interacting with the Technology

One rural generalist suggested that the overall acceptance of telehealth in health services was directly linked to acceptance of teletraining. The increased usage by certain sites meant staff were more comfortable using the technology for other services. Participants from MM 6 locations appeared to have more experience with teletraining and telehealth.

However, for doctors familiar with videoconference technologies, the negative experiences may be dependent on user platforms. Certain platforms were reported to be more successful in facilitating interactive discussion and reducing frustration in terms of logging in. The frustration with using non-user friendly platforms may be amplified because the users are aware of the capabilities of better platforms. Frustrations also arose from limitations to preparation for education sessions, such as availability of PowerPoint presentations ahead of time or testing web links before dissemination.

#### 3.2.3. Work Hours versus Out of Work Hours

A number of doctors mentioned that whilst further education is important and necessary, finding appropriate timings for sessions would be a significant barrier. The main arguments for having training scheduled afterhours were from participants working in hospitals without adequate staff to cover training time absences. Participants who preferred to avoid afterhours training cited reasons such as family commitments and exhaustion after work.

Finding protected time to attend training activities was an ongoing challenge. Participants described similar issues when EM specialists visited for face-to-face training. Interestingly two participants mentioned that if the learning experience is valuable enough, most doctors would reorganise schedules to attend. Yet, both participants worked in a town with a Modified Monash Category of 4, in relatively well-staffed hospitals.

#### 3.2.4. Paid Time versus Own Time

Remuneration for training undertaken outside work time is also an issue. Unfortunately remuneration negotiations are beyond the scope of this paper but may need to be considered if emergency teletraining activities were offered.

#### 3.2.5. Reducing Professional Isolation

Remote participants mentioned that the use of teletraining is an effective strategy for improving professional connection and reducing professional isolation. Seeing colleagues in a similar situation over videoconference enabled participants to feel connected and improved the overall wellbeing of rural doctors.

## 4. Discussion

Most participants in this study had some form of experience with teletraining and identified it as a feasible model of delivering EM education to rural doctors. Of particular note was the Rural Grand Round delivered by video conference for the past seven years, which demonstrates the overall acceptability of this method of training delivery.

Similar to previous studies, some participants agreed that whilst teletraining was not as good as traditional forms of teaching, the ability to have regular education without significant travel made it a useful tool for rural doctors [15, 16].

The findings of this study indicate that rural doctors would like access to peer teaching. Other authors have demonstrated successful use of small group learning via videoconference for medical education, a strategy that would support peer learning [15]. Furthermore its small size would mean logistical considerations such as timings could be overcome by tailoring it to individual rosters. In relation to this study's findings on training needs (reported elsewhere), peer teaching would help rural doctors manage emergencies in the context of limitations specific to rural sites as well as support new provisional fellows taking up senior positions. Furthermore, peer teaching combines both videoconferencing and continued medical education. Both of these factors have been shown to reduce professional isolation and improve retention of rural doctors [8, 17]. However the practicalities in the application of peer teaching require further research.

The doctors in this study raised concerns regarding the use of teletraining for nonstatic forms of learning, unaware that teaching of surgical and EM skills over videoconference has been previously demonstrated [5, 10]. Determining which emergency skills can successfully be taught is an area for future research. However, based on this study's training needs research, procedural skills were not identified as a major training need; therefore skills training via videoconference may not be required. In contrast regular in-house simulations were requested by rural doctors. The possibility of incorporating videoconferencing to simulations was raised by some doctors and rejected by others. A potential avenue that overcomes divided opinion may be a low-fidelity simulation organised via videoconference. Consequently, the technology would not detract from the learning experience and external motivation would ensure it does not get forgotten. However this would require an external organisation to set up such a system. More research would be required before feasibility of simulations with videoconferencing can be confirmed.

Contrary to older studies, technological glitches such as Internet connection were not perceived to be a major concern [4]. This study reaffirms another recent paper on videoconference technologies in North Queensland, suggesting that over time the technology has improved [8]. Instead doctors who were less familiar with videoconferencing were those more resistant to teletraining. Previous studies have found overcoming scepticism and doctors not understanding the value of the technology as important barriers to teleconferencing technology [18]. For some experienced users of teletraining negative experiences may be driven by the inability of the user-platform to meet their expectations.

Opinion was divided about timings for teletraining opportunities. Judging by this variability, perhaps a better approach would be to book teletraining in consultation with the individual site, considering relevant rosters and the personal preference of the doctors. Other logistical concerns such as remuneration were beyond the scope of this paper but may be relevant to training providers/organisational bodies intending to offer teletraining opportunities.

It is of interest that when discussing the limitations of teletraining, participants also discussed the skills of the presenter separate to the technology itself. The successful utilisation of this technology requires alternative teaching styles in comparison to traditional models of lecturing. As mentioned above, interactive sessions are not offered by local training providers, despite having access to the technology. Therefore the feasibility of teletraining may be underreported as training providers are not using the technology to its full capabilities.

## 5. Limitations

The study was limited in its evaluation of the use of videoconferencing to teach EM skills, as most participants had no prior experiences with this learning. Consequently the study's findings are based on participant's assumptions regarding its capabilities.

## 6. Conclusion

Teletraining for management of emergency condition is welcomed and appreciated by rural doctors in North Queensland. For these rural doctors it is a feasible mode of delivering education to rural sites. Among the benefits of teletraining, reducing professional isolation is an important factor for sustaining rural practice. Therefore teletraining models need to be established as core business for health services and training providers including specialist colleges.

## Figures and Tables

**Table 1:** Demographics.

	Junior (11)	Senior (9)
MMM		
1-2	-	3
3	-	-
4	6	4
5	1	1
6	2	1
7	2	-

Postgraduate years/years of fellowship		
0-1	-	2
2	2	-
3	2	1
4	1	1
5	3	
>5	3	5

Advanced skill^*∗*^		
Emergency	1	3
Others	4	4
N/A	6	4

Others		
Medical superintendent	-	1
Involved with training	-	2

^*∗*^Some doctors had more than one advanced skill.

**Table 2:** Teletraining as education.

Subtheme	Quote
Previous experiences with teletraining	“What I actually find more useful [in Rural Grand Rounds] is quite often he gets paediatricians, emergency medicine from [bigger regional centres], from everywhere who actually do speeches and talks” [PF 4]
	“So yeah, that lack of physical-ness about the situation means often you can get apathetic and sometimes just be a bit blasé about it all and sign on just because you have to sign on” [PF 3]
	“Oh, I think it serves a purpose. So these people are spread off all over North Queensland and the distances that are involved are prohibitive for everyone together on a really regular basis” [PF 3]

Face to Face versus videoconference learning	“I think any access to any teaching would be appropriate and even if you do not pick up at come place like do not pick up as much from face to face teaching…Sometimes telehealth teaching can teach you maybe if you do pick up 50% of it just because it's ongoing” [PF 5]
	“I think tele-training is the way of the future especially for people in our area that more and more of us are going out to rural sites” [RR 2]

Peer teaching	“The best benefit is you get to experience different thinking outside the box and different ways of doing things that apply to rural areas, rather than the textbook stuff that does not always apply” [PF 6]
	“I enjoy catching up with people that are in a similar role and have a chance to debrief and have a chance to learn from each other and that is not really a lot of opportunity for that, and the peer education, just people in the same situation and learning from things they have seen and done before, that is probably the only thing I have noticed as a difference” [PF 1]

Teaching skills using videoconference	“I think though that there are certain skills that you need to do person - face-to-face and have immediate feedback on” [RG 2]
	“Unfortunately the technology is what people take away from those learning opportunities; they do not take away the actual message of simulation” [RG 6]
	“There is no reason why they cannot create a resus with a mannequin, put a dress on it and bring it to the ED and run their own simulation. We all have inherent skills and knowledge. Doing simple stuff, it does not need to be complex” [RG 6]
	“The biggest issue with rural is that often you plan to do something every week …then it just falls off the radar… I think you'd need to have a bunch of passionate people at a centralised location to really push it to happen every week” [PF 4]
	“I do not see why you would need someone to teach a practical skill online as a teleconference when you can use YouTube, pause it and revisit all of the steps” [PF 6]

Didactic versus interactive sessions	“Interactive, I definitely prefer, but sometimes in the early years of GP training, a lot of the stuff was a bit didactic, but I did not mind that, I found it to be quiet useful” [GPRNH]
	“There would be scope for that as long as it's not didactic lectures, as long as there can be interaction and the facilitators have a skill in that and the people who are being educated, the students, are prepared enough to interact…it would have to be an interactive thing otherwise you can get that from a YouTube, cannot you?” [RG 3]

[RR] rural reliever; [PHO] GP Registrar Primary House Officer; [GPRNH] non-hospital based GP registrar; [PF] provisional fellow; [FACEM] Fellow of Australasian College of Emergency Medicine; [RG] rural generalist.

**Table 3:** Personal considerations.

Subtheme	Quote
Technological issues	“You could say in theory talk about technological glitches but really they were hardly there in my experience. There is so much support, there's hardly any technological problem” [RG 7]
	I've been to lots of courses where - or video conferences that are halfway through and it's cut out and then they come back and you get a bit of it, and then you've lost the rest of it. So you just end up chatting and not really engaging” [PF 2]

Interacting with the technology	“Places like [Health Service A], Tele-health is what we did. Everyone knew how to work the machines, everyone knew what the limitations of the technologies were. I am not convinced that in [Health Service B] that is the case yet. I think we can still do a little more work to improve our access to utilisation of videoconferencing as a medium of education. I think if you compared the people from [A] and people from [B], I think you would get very different answers. [A] is more tele-health engaged then [B] is” [RG 6]
	“That's something that really frustrates us as people who are really adept at using technology, that they're using platforms that are really not user friendly” [PF 3]

Work hours versus out of work hours	“I find it very hard to imagine squeezing anything into workhours here, because we are so flat out” [PF 6]
	“I've got three kids so often when I come home from work; I am home from work” [PF 4]
	The referral hospital sends an emergency consultant up once every few months to do training. But if it is busy, or you are in theatre you cannot go. So you miss out on that. [PF 1]
	“People tend to make the time [for EMET] because it is such a valuable learning experience” [PF 4]

Paid time versus own time	“Then you've got issues then is that paid or unpaid time, because it's work-related or not work-related…So do you then do extra hours at the hospital, do your kind of video conference and not being paid for them. You know, that that becomes a big thing” [PF 2]
	“SMOs get paid incredible well, they have all these bonuses which I think as long as it is not every night, doing it once a week or once a fortnight they should suck it up” [PF 6]

Reducing professional isolation	“The fact that is keeps you connected with equivalent peers in the region, has been really good” [GPRNH]
	“They are very helpful because more so when you are working in a remote area it's so easy to feel isolated… At least you know that there are other people out there in a similar situation as you. It is really good and it is quite effective”…I think their welfare is important in terms of professional and personal health [RG 7]

[RR] rural reliever; [PHO] GP Registrar Primary House Officer; [GPRNH] non-hospital based GP registrar; [PF] Provisional Fellow; [FACEM] Fellow of Australasian College of Emergency Medicine; [RG] rural generalist.
